# Prevalence of Anemia and Its Associated Factors in Antiretroviral-Treated HIV/AIDS-Positive Adults from 2013 to 2018 at Debre Berhan Referral Hospital, Ethiopia

**DOI:** 10.1155/2020/2513578

**Published:** 2020-03-11

**Authors:** Yared Asmare Aynalem, Wondimeneh Shibabaw Shiferaw, Zeleke Woldiye

**Affiliations:** ^1^College of Health Science, Debre Berhan University, Debre Berhan, Ethiopia; ^2^Debre Berhan Referral Hospital, Debre Berhan, Ethiopia

## Abstract

*Introduction*. Anemia was defined as a hemoglobin level of less than or equal to 13.9 g/dl for male and less than or equal to 12.2 g/dl for female adults. It is one of the most common hematological abnormalities in people living with human immunodeficiency virus/acquired immunodeficiency syndrome (HIV/AIDS) and is a determining factor for disease progression and death. Among the countries in sub-Saharan Africa, Ethiopia is one of the most affected nations by HIV. Therefore, this study aimed to assess the prevalence of anemia and its associated factors among HIV-positive adults that had received antiretroviral treatment (ART) at Debre Berhan Referral Hospital.

**Methods:**

An institution-based, descriptive, cross-sectional study was conducted involving 263 adults with HIV/AIDS that had undergone ART at Debre Berhan Referral Hospital, Ethiopia. Data were collected from patient charts using systematic sampling with a pretested data extraction tool and entered using EpiData 3.1. Variables having a *p* value ≤0.25 in the bivariate were fitted to a multivariable regression model with a 95% confidence interval. *p* value ≤0.25 in the bivariate were fitted to a multivariable regression model with a 95% confidence interval.

**Results:**

Among the 263 HIV-positive patients, 237 (90.11%) were included in the final analysis. The overall prevalence of anemia was 26.2%. Factors that were significantly associated with anemia were past opportunistic infections, patients being in WHO clinical stage III and IV, and a BMI <18.5. Conversely, those patients who took anti-TB medication were less likely to have anemia.

**Conclusion:**

Our study shows that the severity of anemia among HIV/AIDS patients that had undergone ART is lower than most studies conducted in Ethiopia. We also found that opportunistic infection, WHO clinical staging, anti-TB treatment, and low BMI were significantly associated with anemia. Therefore, routine screening of patient nutritional status and opportunistic infections may be useful in predicting and controlling anemia in HIV/AIDS patients.

## 1. Introduction

According to the World Health Organization (WHO), anemia is a condition in which the number or size of red blood cells and, thus, hemoglobin (Hgb) concentration fall below the required level to transport oxygen effectively. It is also defined as a condition in which the Hgb content of the blood is lower than normal for a person's age, gender, and environment, resulting in the reduction of oxygen-carrying capacity of the blood. It affects more than 2 billion people globally, which is ∼30% of the global population. Moreover, anemia is of particular concern for developing countries, where its effects extend beyond human health and significantly impact the social and economic welfare of the nation [1, 2]. It might have a negative effect on quality of life, mortality, morbidity, and socioeconomic progress of a country at large. It affects more developing countries than the developed countries [2]. Based on recent estimates from the World Health Organization (WHO), the prevalence of anemia is 24.8% globally [3].

Anemia is one of the most frequently reported hematological complications in people afflicted with human immunodeficiency virus/acquired deficiency syndrome (HIV/AIDS). Although not well studied, it is estimated that about 60–80% of late-stage HIV patients are anemic. In these patients, 22% of anemia is thought to be caused by HIV treatments including antiretroviral medications [4, 5]. Moreover, anemia is considered one of the key independent predictors of HIV disease progression [6–9]. Even if anemia responds to antiretroviral therapy, it often persists in many patients despite therapy, and such persistent anemia continues to negatively affect prognosis regardless of drug response [10]. Additionally, in asymptomatic HIV/AIDS patients, it is estimated that up to 30% are anemic.

The cause of anemia is multifactorial. For instance, females are more likely to be anemic compared with males worldwide. In a study conducted in the US, it was reported that women had a 71% greater prevalence of anemia than men [4]. Additional contributors to an anemic state include nutritional deficiencies, opportunistic, and parasitic infections, as well as the presence of a chronic infection, which may decrease erythropoiesis [11]. Some of the factors associated with anemia in HIV/AIDS patients in Ethiopia are a low CD4 count, WHO clinical stage III and IV, opportunistic infections (OIs), and a low BMI [12–15].

Ethiopia is ranked as one of the countries with the highest HIV burden worldwide, with about 1.1 million people living with HIV and about 14,405 new HIV infections being reported in 2016 [16]; anemia was a major comorbidity in many of these cases [13, 14, 17]. In Ethiopia, despite the availability of HAART for more than 13 years, there is still substandard data on the prevalence of anemia and its associated factors in HIV-positive adults. Therefore, this study aims to assess the prevalence of anemia and its associated factors among HIV-positive patients that have received antiretroviral treatment (ART) at Debre Berhan Referral Hospital (DBRH), Ethiopia, from 2013 to 2018.

## 2. Materials and Methods

### 2.1. Study Design, Area, Period, and Populations

This study was an institution-based, descriptive, cross-sectional study involving HIV-positive adults that had received ART from April to March 2019. The study was conducted in DBRH, North Shewa Zone, Amhara, Ethiopia. Based on the hospital medical record report, the total number of HIV-positive individuals that had received ART from 2013 to 2018 was 847 (285 males and 562 females). All HIV-positive individuals who had received ART and were aged 18 years or older were our source population, and all HIV-positive individuals who had received ART, were aged 18 years or older, and had followed up at DBRH from 2013 to 2018 were our study population.

### 2.2. Eligibility Criteria

Individuals were considered eligible if they were aged 18 years or older, had a confirmed HIV infection upon follow-up at DBRH, and had a detailed history and laboratory results such as Hgb and CD4 counts. Those individuals that did not have a detailed history and laboratory results and patients with incomplete records were excluded.

### 2.3. Sample Size Determination and Sampling Technique and Procedure

The sample size was calculated using the single population proportion sampling formula with the following assumptions: 5% marginal error, Z is the value corresponding to the 95% confidence interval (CI) = 1.96, and proportion of anemia (P) = 53.6% [18], which was taken from a study at Arba Minch, Ethiopia. Using these parameters, the sample size was found to be 382. Because the source population was less than 10,000, we used the correction formula to determine the final sample size of 263. Patient chart numbers were taken from the HIV/AIDS registration book. The systematic random sampling technique was employed to identify the study units using the ART unique numbers from the registration books.

### 2.4. Study Variable

#### 2.4.1. Dependent Variable

Prevalence of anemia in HIV-positive adults that had received ART.

#### 2.4.2. Independent Variables

*(1) Sociodemographic Characteristics*. Age, sex, marital status and occupation of mother or caregiver, residence, parental status (alive or dead), and family size.

*(2) Baseline Clinical and Laboratory Information*. CD4 count, Hgb, past opportunistic illnesses, tuberculosis (TB) test, WHO staging, BMI, developmental or functional history, and treatment.

*(3) ART and Other Medication Information*. Regimen at follow-up, adherence to ART, cotrimoxazole preventive therapy, side effects of ART drugs, regimen change, and regimen stopped.

### 2.5. Data Collection Methods and Tools

The data tool was developed from the standardized ART entry and follow-up form currently used by ART clinics. It was also developed by referring to the relevant literature. The available information on the patient records was first observed, and an appropriate data extraction format was prepared in English and then was translated into Amharic. The data were then collected by the principal investigators using the prepared data collection format on the already existing records. Analysis of serum Hb levels was a routine laboratory investigation. Therefore, it was collected from the laboratory results done and documented at the time of data collection or done recently, before two weeks back.

### 2.6. Data Quality Assurance

Data quality was assured by designing a proper data collection checklist. The checklist was evaluated by experienced researchers. The data collection instrument was tested on 5% of the sample size. Language clarity, appropriateness of data collection tools, estimate of the time required, and the necessary amendments were considered based on the test. During data collection, close supervision and monitoring was carried out by supervisors and the investigator to ensure the quality of the data. Finally, all of the collected data were checked by the supervisor and the investigator for its completeness and consistency during data management, storage, and analysis. Consistency was examined through random selection of checklist. Accuracy of the data was also assured by everyday on-site cross-checks during data collection.

### 2.7. Data Processing and Analysis

Data were cleaned, edited, and coded. Any errors were identified and corrected after review of the original data using the code numbers. The data were then entered using EpiData version 3.1 and analyzed using SPSS 24 statistical software. Anemia was defined based on hematological reference values for the adult Ethiopian population [19]. Consequently, it was defined as an Hgb ≤13.9 g/dl for males and ≤12.2 g/dl for females. It was also categorized into mild (10–12.2 g/dl for females and 10–13.9 g/dl for males), moderate (8–10 g/dl), and severe (<8 g/dl) for both sexes. Descriptive statistics were used to present the sociodemographic and other anemia-related factors. In the ultimate multivariable models, the level of multicollinearity was checked and fitted using variance inflation factor and tolerance and found within a tolerable range (all variables of the variance inflation factor value were <3.2) and tolerance (all variable values > 0.2). The binary regression model assumption was checked using a goodness test such as the Hosmer–Lemeshow goodness of fit, and variables having *p* values >0.05 were considered as fulfilling the assumption. The bivariate regression model was fitted for each explanatory variable. Accordingly, those variables having *p* values ≤0.25 in the bivariate analysis were fitted to the multivariable regression model with 95% CI. *p* values ≤0.05 were considered statistically significant in the multivariate analysis.

## 3. Results

### 3.1. Sociodemographic Characteristics of the Study Participants

Of the 263 HIV-positive adult patient records reviewed, 237 (90.11%) were included in the final analysis. Around two-thirds (67.9%) of these participants were females, and the majority of them (83.5%) came from an urban area. Half of the study participants were married. The mean age of the study participants was 35 years old, and the minimum and maximum ages were 18 and 71 years old, respectively. Among the eligible patients, 92 (38.8%) were 15–30 years old, 110 (46.45%) were 31–45 years old, and 35 (14.8%) were older than 45 years old (Table 1).

### 3.2. Baseline Clinical, Laboratory, and ART Information

Among the 237 participants, 88 (37.1%) had past opportunistic infections. Of these individuals, 25 (28.4%) had candidiasis, 18 (20.5%) had pneumonia, and 16 (18.2%) had herpes zoster. Additionally, the majority of the patients (227 (95.8%)) were functional and working and more than half of the participants were at WHO clinical stage I. More than half of the study participants also had a BMI <18.5 kg/m^2^ (Table 2).

### 3.3. RT Treatment and Other Medication-Related Characteristics of the Participants

Only 45.3% of the participants had a history of regimen change throughout the entire follow-up. From those who had a history of regimen change, toxicity or drug side effects (54.8%) is the most common reason for the alteration, with drugs being out of stock (24.6%) and clinical failure (9.8%) also being prevalent within the group. In addition, among those who had side effects from the drug (17.84%) during follow-up, anemia (30.8%) was found to be the most common complaint followed by nausea (20.8%) and rash (12.3%). Almost all study participants received cotrimoxazole prophylaxis (223 (94.1%)), and zidovudine-lamivudine-efavirenz (AZT-3TC-EFV) was given to 228 (96.2%) of the patients at follow-up (Table 3).

### 3.4. Prevalence of Anemia among HIV-Positive Patients

**Figure 1:**
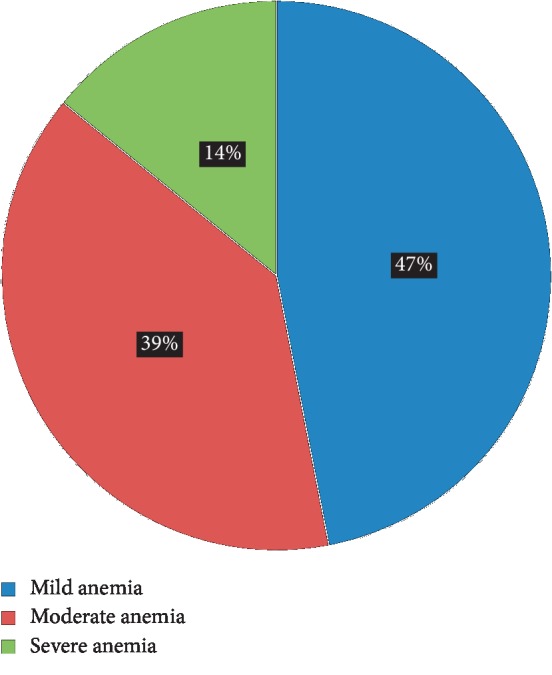
Hgb classification of the participants from the DBRH ART clinic who were on HAART.

The prevalence of anemia among HIV-positive adults on HAART was 26.2% (62/237). Of those, 47% and 39% had mild and moderate anemia, respectively (Figure 1).

### 3.5. Anemia-Associated Factors among HIV-Positive Patients on HAART

Both the bivariate and multivariate logistic regressions were done to assess the anemia-associated factors among HIV-positive patients. Our bivariate analysis showed that factors including past OIs, WHO clinical staging (stage III and IV), BMI, previous anti-TB treatment, and OI during follow-up were associated with anemia. Moreover, in our multivariate logistics analysis model, only having a past OI, WHO clinical staging (stage III and IV), previous anti-TB treatment, and OI were associated with anemia. Multivariate analysis revealed that patients who had an OI were 1.6 times more likely to develop anemia compared with those who did not have an OI (AOR: 1.6 (95% CI: 1.59–4.33)). Patients at WHO clinical stage III and IV were almost three times more likely to die compared with those at WHO clinical stage I and II (AOR: 2.98 (95% CI: 1.25–7.12)). In this study, patients who had taken anti-TB medication were 80% less likely to have anemia than those patients who did not take anti-TB medication (AOR: 0.2 (95% CI: 0.12–0.91)). Moreover, HIV-positive patients who had a BMI ≤ 18 were three times more likely to have anemia than those individuals having a BMI ≥ 25 (AOR: 3.1 (95% CI: 1.33–7.26)) (Table 4).

## 4. Discussion

Anemia is a major concern for those individuals who are suffering from HIV/AIDS as it is a significant determinant of disease progression. This study showed that 26.2% of HIV-positive adults that were treated at DBRH from 2013 to 2018 had anemia. This is consistent with the results from studies in Rwanda (29%) [20], Nigeria (24.3%) [6], and South Africa (25.8%) [21]. However, the prevalence of anemia in HIV-positive patients was lower in this study than in studies conducted in other areas of Ethiopia including Debre Tabor (34%) [15], Arba Minch (53.6%) [18], Gondar (35%) [7], and Zewditu Memorial Hospital (42.9%) [14]. Similarly, our results were much lower than those found in studies of patients from Nigeria (60.61%) [22], Tanzania (56%) [23], Bayamón, Puerto Rico (41%) [24], Nepal (55.8%) [25], and China (39.2%) [26]. However, the overall prevalence of anemia among HIV-positive adults was higher than that found in patients from Jimma (23.1%) [8], India (16.2%) [9], and Ghana (23.8%) [27]. In this study, the prevalence of mild, moderate, and severe anemia was 47%, 39%, and 14%, respectively, which is lower than the results presented from a study in China [26]. These differences in the burden of anemia among HIV-positive patients may be related to differences in the sample size (e.g., sample size was large in Nigeria), sociodemographic disparity, characteristics of the study participants, and changes in treatment modalities. Additionally, all participants in this study were HIV-positive patients on ART. This might also indirectly show the efficacy of HAART in reducing HIV-associated anemia by reducing the incidence of OIs, chronic illnesses, and improving the nutritional status of patients.

In this study, OI, WHO clinical staging, low BMI, and anti-TB treatment were significantly associated with anemia. Indeed, advanced WHO clinical staging (III and IV) was found to be an independent associated factor of anemia for patients with HIV/AIDS. Adults with advanced WHO clinical stage (III and IV) HIV/AIDS at the time of ART initiation were almost three times less likely to have anemia compared with those in WHO clinical disease stage I and II at the time of ART initiation. This is supported by a previous study conducted in Ethiopia [28].

In this study, the odds of getting anemia among HIV-positive patients that had a BMI ≤18 were three times higher than those individuals having a BMI ≥25. This is in line with a study from Rwanda [29]. The association between anemia and low BMI may stem from the fact that HIV/AIDS causes malnutrition in many individuals due to reduced appetite, OIs, and treatment side effects. This might lead to difficulty in ingestion and diarrhea, which might limit nutrient uptake including iron.

This study also noted that the likelihood of having anemia was higher among patients with OIs. HIV-positive adults with OIs were 1.6 times more likely to have anemia compared with patients with no OI. This is supported by a previous study done in Jimma hospital [8]. This could be due the fact that even if ART is initiated for all HIV-positive patients as early as possible, most patients present for care and treatment at late clinical stages. Therefore, they might be diagnosed as having OI, which are the major causes of morbidity and mortality including anemia among HIV-infected patients.

Another anemia-associated factor for HIV-positive patients found in this study was anti-TB treatment. Adults taking an anti-TB treatment at the time of ART initiation were 80% less likely to have anemia compared with those who were not taking an anti-TB treatment. This is supported by a study done in Ethiopia and Tanzania [23, 30]. This association between anemia and anti-TB treatment may be due to the fact that TB is the most frequent life-threatening OI and leading cause of death among HIV-infected people. It increases HIV replication through the process of immune activation, which leads to an increased viral load. Thus, it is vital for both TB and HIV control programs to be synergistic to alleviate the double burden of TB/HIV in populations affected by both diseases. Moreover, TB management programs might be improved if they were able to reduce anemia in their coinfected patients.

## 5. Conclusions

This study showed that the prevalence of anemia in HIV/AIDS patients is lower than that in most studies conducted in Ethiopia. We also found that the presence of OIs, WHO clinical stage III and IV, low BMI, and anti-TB treatment were significant factors associated with anemia in these patients. Therefore, the government needs to focus on repetitive screening of HIV-infected patients with low Hgb levels and had opportunistic infection and strengthen appropriate treatment of TB patients and nutritional diversification.

## Figures and Tables

**Table 1:** Sociodemographic characteristics of patients.

Characteristics		Frequency	Percentage
Sex	Male	76	32.1
	Female	161	67.9
Age	18–30	92	38.8
	31–45	110	46.4
	>45	35	14.8
Residence	Urban	198	83.5
	Rural	39	16.5
Marital status	Single	29	12.2
	Married	123	51.9
	Divorced	56	23.6
	Widowed	29	12.2

**Table 2:** Baseline clinical and laboratory information of patients.

Characteristics			Frequency	Percentage
Past opportunistic infection	Herpes zoster		16	18.2
	Candidiasis (oral and esophageal)		25	28.4
	*Pneumocystis* pneumonia		18	20.5
	Past TB test	Positive	27	11.4
		Negative	210	88.6
	Diarrhea		11	12.5
	Others		18	20.3
Functional status	Working		227	95.8
	Ambulatory		10	—
WHO clinical staging	Stage I		146	61.6
	Stage II		43	18.1
	Stage III and IV		48	20.3
CD4 counts	<350		74	31.2
	≥350		163	68.8
BMI (kg/m^2^)	<18.5		66	27.8
	18.5–24.9		123	51.9
	≥25.0		48	20.3

**Table 3:** ART treatment and other medication-related characteristics of patients.

Characteristics		Frequency	Percentage

ART eligibility criteria	CD4 < 350 and WHO stage IV	16	6.8
	WHO stage II and III	26	10.9
	Without criteria	195	82.3
OI prophylaxis	Yes	225	94.9
	No	12	—
ART side effects during follow-up	Yes	130	54.8
	No	107	45.2
Regimen change during follow-up	Yes	103	43.3
	No	134	56.7

**Table 4:** Bivariate and multivariate logistics regression analysis of variables.

Variables		Anemia		COR (95% CI)	AOR (95% CI)
		Non-anemic	Anemic		
Sex	Male	21	55	Reference	
	Female	42	119	2.1 (0.84–3.68)	1.16 (0.23–2.50)
OIs	Yes	37	50	0.3 (0.15–0.51)^*∗*^	1.6 (1.59–4.33)^*∗*^
	No	26	124	Reference	
WHO staging	Stage I and II	36	12	Reference	
	Stage III and IV	27	162	0.5 (0.2–0.89)^*∗*^	2.9 (1.25–7.12)^*∗*^
Anti-TB Rx	Yes	25	24	Reference	
	No	38	150	0.4 (0.1–1.11)^*∗*^	0.2 (0.12–0.91)^*∗*^
BMI	<18	24	42	3.24 (1.63–6.45)	3.1 (1.33–7.26)^*∗*^
	18.5–24.9	22	101	1.2 (0.42–3.03)^*∗*^	1.3 (0.43–3.73)
	≥25	17	31	Reference	

^*∗*^Indicates significance at a *p* value <0.05.

## Data Availability

The data are accessible from the corresponding author upon reasonable request.

## Abbreviations

AIDS: Acquired immunodeficiency syndrome
AZT: Zidovudine
DBRH: Debre Berhan Referral Hospital
DBU: Debre Berhan University
HAART: Highly active antiretroviral therapy
Hgb: Hemoglobin
HIV: Human immunodeficiency virus
OIs: Opportunistic infections
PLWHA: People living with HIV/AIDS
SPSS: Statistical package for social science
WHO: World Health Organization.
